# Filament stretching during micro-extrusion of silver pastes enables an improved fine-line silicon solar cell metallization

**DOI:** 10.1038/s41598-022-16249-5

**Published:** 2022-07-19

**Authors:** Katharina Gensowski, Maximilian Much, Elisabeth Bujnoch, Stefan Spahn, Sebastian Tepner, Florian Clement

**Affiliations:** Fraunhofer Institute for Solar Energy Systems, Heidenhofstraße 2, 79110 Freiburg im Breisgau, Germany

**Keywords:** Engineering, Materials science, Physics

## Abstract

The metallization of heterojunction solar cells requires a further reduction of silver consumption to lower production costs and save resources. This article presents how filament stretching of polymer-based low-temperature curing Ag pastes during micro-extrusion enables this reduction while at the same time offering a high production throughput potential. In a series of experiments the relationship between the printing velocity and the filament stretching, thus the reduction of Ag-electrode widths and Ag laydown is evaluated. Furthermore, an existing filament stretching model for the parallel dispensing process is advanced further and utilized to calculate the elongational viscosity. The stretching effect enables a reduction of the Ag-electrode width by down to Δw_f_ = − 40%_rel._ depending on the nozzle diameter and paste type. The Ag laydown has been reduced from m_Ag,cal._ = 0.84 mg per printed line to only m_Ag,cal._ = 0.54 mg per printed Ag-electrode when 30 µm nozzle openings are used, demonstrating the promising potential of parallel dispensing technology for the metallization of silicon heterojunction solar cells.

## Introduction

The International Technology Roadmap for Photovoltaic (ITRPV) predicts a world market share of silicon heterojunction (SHJ) solar cells of 10% in 2024 and 17% in 2030 which corresponds to a substantial rise compared to 3% in 2019^1^. In the last 15 years, several research groups worked towards a further reduction of the Ag-electrode width *w*_*f*_ and Ag laydown per cell *m*_*Ag*_ to save silver, thus further minimizing cell production costs. Lorenz et al. illustrated this trend for flatbed screen-printed Ag-electrodes (in photovoltaic industry referred to as ‘fingers’) and indicated that intense industrial optimization of pastes, screens and machine technology were the main reasons for decreasing the Ag-electrode width over the years^2^. In 2020, Tepner et al. presented a flatbed screen-printed line electrode with a width of w_f_ = 19 µm and an electrode height of h_f_ = 18 µm on a passivated emitter and rear cell (PERC)^3^. Besides the decrease in Ag-electrode widths, the ITRPV predicts a total silver consumption of only 50 mg silver per cell in 2030^1,4^. In order to achieve that, the parallel dispensing technology as an alternative printing process has emerged in recent years. Pospischil et al. demonstrated a dispensed line electrode with a width of w_f_ = 17 µm on a PERC solar cell. In that study, they showed that the Ag laydown as well as the electrical cell performance were improved compared to the reference^5–9^. These publications show an impressively successful development over the last years for PERC metallization.

However, the state of research and development of low-temperature curing Ag pastes for SHJ solar cell metallization is far from those results, especially regarding obtainable process velocities and achievable Ag-electrode widths. High throughput rates and low silver consumption per SHJ cell are requirements to increase the market share of this high-efficiency solar cell concept. Erath et al. recently published applicable flooding and printing velocities of up to v = 400 mm s^−1^ for flatbed screen printing^10^. Descoeudres et al. presented a w_f_ = 16 µm wide, screen-printed Ag-electrode by using a special, knotless screen with screen openings of w_n_ = 12 µm^11^. Our latest results for SHJ metallization by parallel dispensing showed optimized line electrode widths of w_f_ = 34 µm and an increased optical aspect ratio of AR_o_ = 0.55 when using 25 µm nozzle openings. In that study, the Ag laydown of a 156 mm single line was m_Ag_ = 0.30 mg electrode^−1^^12^. To achieve further progress in low-temperature curing Ag pastes, an understanding of the paste’s inner state during micro-extrusion is needed to solve the limitations of applicable process velocities and line electrode widths. Until now, the reduction of Ag-electrode widths is limited because the paste shows a significant spreading tendency, thus further adaptation of the formulations is potentially required.

However, one way to achieve narrow Ag-electrode shapes may be the utilization of filament stretching during micro-extrusion^13,14^. In this study, we present a detailed description and experimental evaluation on how the paste threads are stretched in uniaxial dimension, resulting in significant necking and therefore reducing the thread diameter before getting in contact with the substrate. Usually, to quantify this effect, extensional properties of fluids are determined for uniaxial extension by capillary breakup extensional rheometer (CaBER)^15,16^ or filament stretching extensional rheometer (FiSER)^17–19^. At this point, no reliable methods to determine the extensional properties of highly filled yield stress pastes are known, therefore, we analyze the impact of this phenomenon by using the dispensing process itself. We evaluate two low-temperature curing Ag pastes regarding the maximal obtainable process velocity and its correlation to the electrode geometry. For that, we implement a variation of nozzle openings (25 µm ≤ *D* ≤ 45 µm) and process velocities (50 mm s^−1^ ≤ *v*_*process*_ ≤ 500 mm s^−1^) and measure the Ag laydown and printed Ag-electrode widths to quantify the filament stretching effect. Further, we determine the paste’s rheological properties in shear and elongation and correlate the data to the effects observed during printing.

## Theoretical background

This paragraph summarizes the state-of-the-art literature regarding the behavior of paste threads during micro-extrusion. Clasen et al. presented the complexity of dispensing fluids with a rather intricate rheology by describing the ‘map of misery’. The ‘map of misery’ includes nondimensional numbers, e.g. Ohnesorge number *Oh*, Elasto-Capillary number *E*_*c*_ and Intrinsic Deborah number *De*_*o*_, which describe the relationship between various material properties. Further characterization can be accomplished through dynamic nondimensional numbers such as capillary number *Ca*, Weber number *We* and Weissenberg number *Wi*^20^. Whenever a fluid is extruded through a nozzle, the relationship between these parameters dictate the physical evolution of the fluid after the nozzle exit. For suspensions, e.g. pastes, the thread may decrease its diameter over time due to its increasing weight. This thinning of the paste thread is mainly reinforced by the surface tension and balanced by the resisting force. It can be differentiated between viscosity-controlled thinning^21^, inertia-controlled thinning^22,23^ and elasticity-controlled thinning^24^.

**Figure 1:**
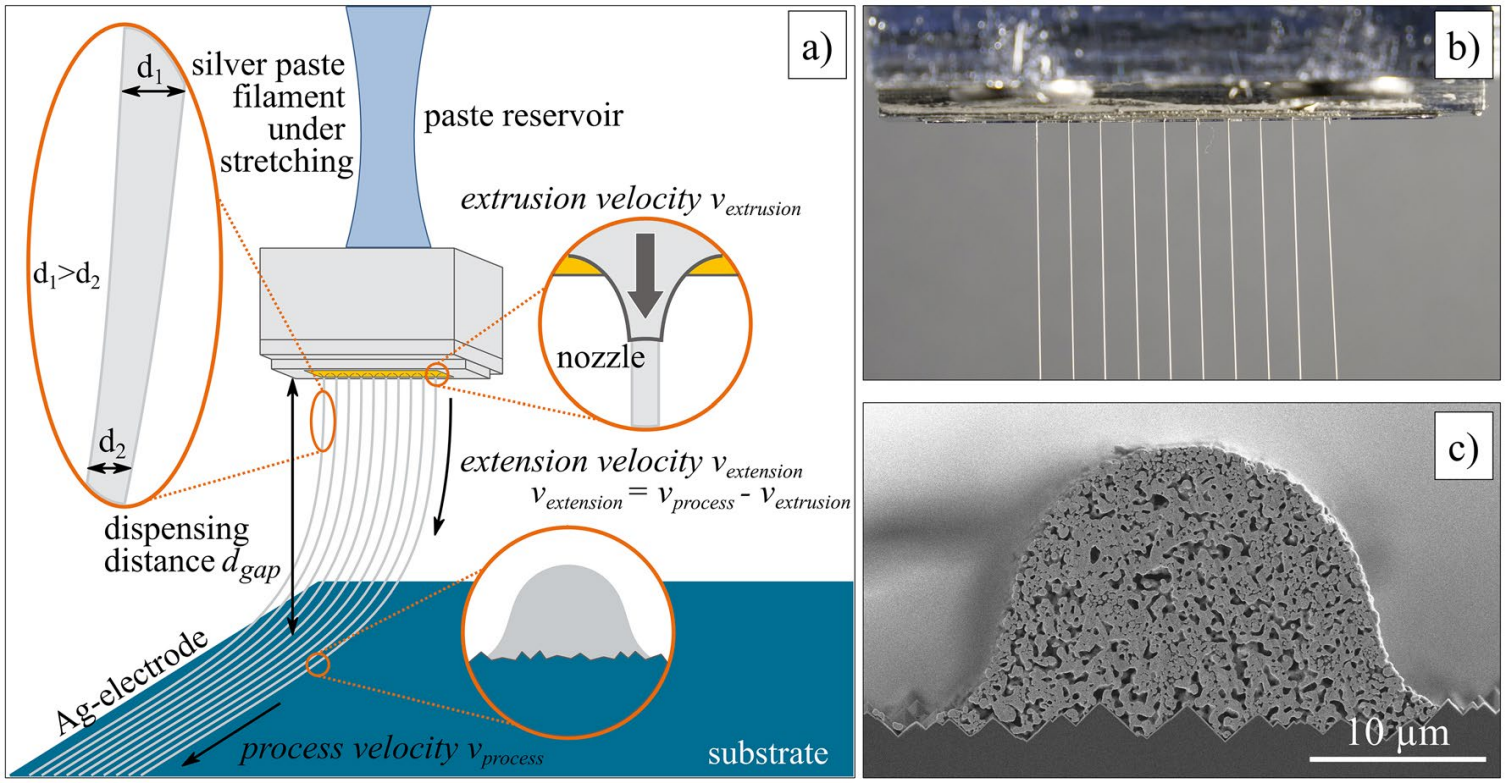
Overview on the parallel dispensing approach for solar cell metallization. (**a**) Illustration of filament stretching during micro-extrusion of low-temperature curing Ag pastes. The print head is positioned above the substrate with the dispensing gap *d*_*gap*_; in this gap the filament stretching occurs. The paste threads are stretched from the initial diameter *d*_*1*_ to a smaller diameter *d*_*2*_. The extensional velocity *v*_*extension*_ is determined by the difference in process velocity *v*_*process*_ and the extrusion velocity *v*_*extrusion*_ (figure according to^13^). (**b**) Simultaneous extrusion of ten paste threads through 35 µm nozzle openings. A uniform paste distribution inside the print head ensures a homogeneous paste extrusion^8,25^. (**c**) Scanning electron microscope (SEM) image of a dispensed Ag-electrode (cross-sectional view). Here, a low-temperature curing Ag paste was applied onto SHJ solar cell by parallel dispensing. The SEM image is taken out of ref.^12^.

Kunpai et al. demonstrated the filament thinning of Ag paste formulations during micro-extrusion caused by different amounts of graphite nanofibres. The widths of the dispensed structures were smaller than the applied nozzle openings because of the elongational properties of the paste^13^. Figure 1 depicts the stretching of paste threads in a micro-extrusion process using a parallel print head developed at Fraunhofer ISE^25^. Here, all paste threads exit the nozzle outlet simultaneously^8^. To describe the filament stretching in more detail, three distinguished velocities are defined. The paste flows from a paste reservoir through the print head and finally through the nozzle opening with an extrusion velocity *v*_*extrusion*_. The extrusion velocity *v*_*extrusion*_ depends on the paste formulation and subsequently the rheological behavior as well as the process pressure *p*, the process temperature *T* and the nozzle diameter *D*. The process velocity *v*_*process*_ defines the pace at which the substrate moves underneath the print head, mainly dictating the throughput rate of the metallization process. The specific range for process velocities that allow a stable and homogeneous printing result depends on the same influencing factors as the extrusion velocity *v*_*extrusion*_ and additionally on the dispensing gap *d*_*gap*_. The extruded paste threads are free-hanging between the nozzle outlet and the substrate, thus hanging within the dispensing gap. The paste threads are then stretched, which means that the diameter of the paste threads constantly decreases from the nozzle outlet to the contact point of the paste on the substrate. The length of these paste threads can be quantified by the parameter characteristic length *l*_*c*_^13^. The wetting behavior of the strengthened paste threads onto the substrate is not considered in Kunpai’s modeling approach. However, especially the impact of textured surfaces like solar cell substrates might be significant. The extensional velocity *v*_*extension*_ is the difference between the process velocity *v*_*process*_ and the extrusion velocity *v*_*extrusion*_, hence the filament stretching can be enhanced by increasing the process velocity *v*_*process*_ or decreasing the extrusion velocity *v*_*extrusion*_ in relation to each other.

A significant filament stretching requires sufficient elongation properties of the extruded suspension, therefore a high Hencky deformation as well as a high Hencky deformation rate. The elongational viscosity for uniaxial extension is estimated by CaBER^15,17,26,27^ or FiSER^19^. The uniaxial extensional deformation corresponds to the flow of the paste thread during the dispensing process. The suspension properties in extensional flows and in shear flows can differ significantly, as shown in several publications for polymer solutions or polymer melts. Further publications show results for extensional rheological characterization of complex suspension with significant yield stress^28–30^.

A cylindrical geometry of the sample with a length *L* and a diameter *D* is assumed for determining the elongation properties. The Hencky strain *ε* is defined in Eq. (1), whereby *L*_*0*_ is the initial length and *D*_*0*_ the original diameter, respectively.

1 $$\varepsilon =\mathrm{ln}\frac{L}{{L}_{0}}=2\mathrm{ln}\frac{D}{{D}_{o}}$$

The change in diameter of the cylindrical sample geometry over time is defined as Hencky deformation rate $\dot{\varepsilon }$ (Eq. (2)).

2 $$\dot{\varepsilon }(t)= -\frac{2}{D}\frac{dD}{dt}$$

Schuemmer and Tebel define the uniaxial elongational viscosity *η*_*e*_ as follows in Eq. (3), whereby *σ*_*zz*_ is the axial normal stress in the fluid filament and *σ*_*rr*_ is the radial normal stress^16,31^.

3 $${\eta }_{e}(t)= \frac{{\sigma }_{zz}- {\sigma }_{rr}}{\dot{\varepsilon }(t)}$$

The normal stresses *σ*_*zz*_ and *σ*_*rr*_ are defined as follows in Eqs. (4) and (5), where *Γ* is the surface tension and *F* is the axial force inside the filament.

4 $${\sigma }_{zz}= \frac{4 (F- \pi \Gamma D)}{\pi D^{2}}$$

5 $${\sigma }_{rr}= -\frac{2\Gamma }{ D}$$

Using the definition of the elongational viscosity (see Eq. (3)) in combination with the equations of the normal stresses (see Eqs. (4) and (5)) and the Hencky deformation rate (Eq. (2)), results in the elongational viscosity *η*_*e*_ in Eq. (6).

6 $${\eta }_{e}(\mathrm{t})= \frac{\Gamma }{dD/dt}- \frac{2F}{\pi D dD/dt}$$

When *σ*_*zz*_ = 0 is assumed, Eq. (6) simplifies to the apparent elongational viscosity *η*_*e,app*_ in Eq. (7). This assumption for the axial normal stress is necessary in CaBER experiments because the axial force *F* in the filament cannot be determined^26^.

7 $${\eta }_{e,app}(\mathrm{t})= -\frac{\Gamma }{dD/dt}$$

## Experimental section

In this study, two different low-temperature curing Ag pastes A and B are analyzed regarding their rheological properties in shear and elongation as well as their printability in the dispensing process. The highly filled suspensions are developed to be applied onto transparent conducting oxide layers of SHJ solar cells and thus require a curing temperature of T_c_ = 200 °C to form contacts with low contact resistivity. The paste formulations consist of solvents, an epoxy-phenolic resin system, the same proportion of spherical silver nano-powders and polymer-based additives, if necessary. The solvent mixture is made up of 2-phenoxyethanol and 2-(2-hexoxyethoxy)ethanol. Above 90 wt% of the paste components are nonvolatile after the curing process.

### Rheological characterization of low-temperature curing Ag pastes

The shear viscosity and the thixotropy of the low-temperature curing Ag pastes A and B are measured with the commercial rotational rheometer MCR 702 using the TwinDrive technology by Anton Paar GmbH, Germany. A parallel plate geometry with a diameter of D = 25 mm and a roughness of R_q_ = 2–4 µm is used. The roughened surface of the geometries should reduce the wall slip during the characterization of highly filled suspensions, however, the roughed surface cannot completely suppress this phenomenon^32^. After applying the sample onto the bottom plate geometry, the upper plate moves down to a gap distance of d_trim_ = 0.235 mm. Following, the excess suspension at the geometry edges is removed. The measuring gap between the upper and bottom plate is d_measure_ = 0.2 mm, the measuring temperature is set to T = 25 °C. A waiting time of t = 5 min is chosen before each beginning of the measurement to achieve a homogeneous temperature distribution within the sample and to recover the inner structure of the suspension. To determine the shear viscosity, a stepwise controlled shear rate mode between $\dot{\gamma }$ = 10^–2^ s^−1^ to $\dot{\gamma }$ = 10^4^ s^−1^ is applied. The measuring time per measuring point is reduced logarithmically from t = 55 s to t = 0.3 s. In total, 45 measuring points are measured. The thixotropic behavior of the highly filled suspension is estimated using the ‘three interval thixotropy test (3ITT)’^33^. The 3ITT method is well described in the literature^34–36^. The measuring profile is divided into three intervals whereby the frequency is kept constant at f = 1 Hz. In the first and third interval, the paste is charged at a constant low deformation with an oscillating vibration amplitude of γ = 0.1%. In this experiment, 30 measuring points are chosen with a constant measuring time of t = 10 s for the first interval and 40 measuring points with a constant measuring time of t = 15 s for each measuring point for the third interval. The third interval corresponds to the reconstruction behavior of the fluid. In the second interval, a deformation of γ = 80% is applied. The measuring time for each measuring point is set to t = 5 s for a total of 60 measuring points. In addition, the yield stress of both pastes is determined by an impeller setup. Here, the measuring gap is set to d_measure_ = 0.3 mm and a shear stress between τ = 10^–2^ Pa to τ = 5·10^3^ Pa is applied. The measuring time for each measuring point is set to t = 5 s. The yield stress is determined by the tangent intersection point method^32,37^.

At least three independent measurements are performed for each configuration. The diagrams depict the mean values of all measurement repetitions and their standard deviations as error bars. Each measurement is conducted by using a new sample. The measuring profiles of the shear rheological characterization are defined based on preliminary tests and literature that analyze also highly filled suspensions^32,38,39^. A high-speed imaging camera observes the paste during the rotational rheometry measurement for detecting potential errors and observing wall slip effects.

**Figure 2:**
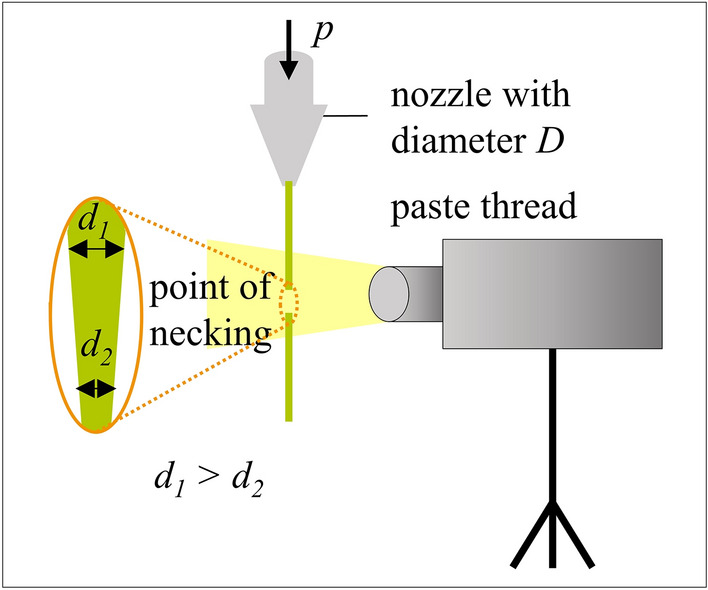
Experimental setup of the rheological experiment to determine the time-dependent diameter of the extruded filament.

The second rheological experiment focuses on the determination of elongation properties of pastes A and B. The samples are extruded through a single nozzle with diameters of D = 110 µm, D = 160 µm and D = 230 µm. These micro-precision needles from Vieweg GmbH, Germany, have conical-shaped tips and are made of a nickel-silver alloy. The applied process pressure *p* is preset to a constant value. The time-dependent change of the diameter of the paste thread is determined by using high-speed imaging. For that, the high-speed imaging camera IDT OS7 with 2000 to 2500 frames per second is placed in front of the experimental setup and focused on the point of necking (see Fig. 2). At least four independent measurements are performed. Further, the high-speed imaging camera is used to determine the extrusion velocity *v*_*extrusion*_ for nozzle openings of D = 25 µm to D = 45 µm by using our ‘GECKO’ R&D print head^25^. This print head extrudes ten paste threads simultaneously. The geometric design of our R&D print head is based on computational fluid dynamics simulations to guarantee a homogeneous paste distribution inside the print head^6,8^. The applied process pressure *p* is preset to the equal constant value used for the dispensing tests onto substrates. The extrusion velocity *v*_*extrusion*_ of each paste thread is calculated by tracking the pixel movement per time increment. Three independent measurements are conducted.

### Parallel dispensing and characterization of printed line electrodes

In this experiment, we have conducted a series of experiments to evaluate the process velocity ranges of two different low-temperature curing Ag pastes. Both pastes are extruded through nozzle openings of D = 45 µm, D = 40 µm, D = 35 µm, D = 30 µm and D = 25 µm. The process pressure *p* and the dispensing gap d_gap_ are kept constant at d_gap_ = 250 µm for all experiments. The so-called ‘GECKO’ R&D print head is used to perform all dispensing experiments^25^. Furthermore, a commercial table robot is used as described in literature^40^. The process velocity *v*_*process*_ is varied between v_process_ = 50 mm s^−1^ and v_process_ = 500 mm s^−1^ in 10 mm s^−1^ increments for each nozzle diameter and paste combination. Each parameter combination is repeated independently three times. After applying low-temperature curing Ag pastes on the substrate, a curing process is conducted in a convection oven R0400FC from Essemtec AG, Switzerland. A curing temperature of T_c_ = 200 °C for a curing duration of t_c_ = 5 min is applied for all samples.

After the curing step, the electrode shapes are visually evaluated regarding their homogeneity. Homogenous, straight lines of category II are characterized by using the 3D confocal laser scanning microscope OLS4000 from OLYMPUS with a magnification of 50x. Nine measurements are performed for each parameter combination. The microscope images are analyzed by the Fraunhofer ISE software, the so-called ‘Dash’^41^. In this case, the electrode widths *w*_*shading*_ and *w*_*core*_, the maximal electrode height *h*_*f,max*_ as well as the cross-sectional area *A*_*cross*_ are determined. The shading electrode width *w*_*shading*_ is defined as the maximum electrode width, including any paste spreading. The core electrode width w_core_ is this part of the shading electrode width which shows an apparent electrode height and thus significantly impacts the lateral electrode resistance. Based on these values, the optical aspect ratio *AR*_*o*_ and the spreading coefficient *ζ*_*spreading*_ are calculated as given in Eqs. (8) and (9)^6,39^. These geometrical parameters that describe an electrode shape are visualized in a SEM image in ref.^39^.

8 $${AR}_{o}= \frac{{h}_{f,max}}{{w}_{shading}}$$

9 $${\zeta }_{spreading}= \frac{{w}_{core}}{{w}_{shading}}$$

## Results and discussion

### Rheology of low-temperature curing Ag pastes

#### Behavior under shear flow

**Figure 3:**
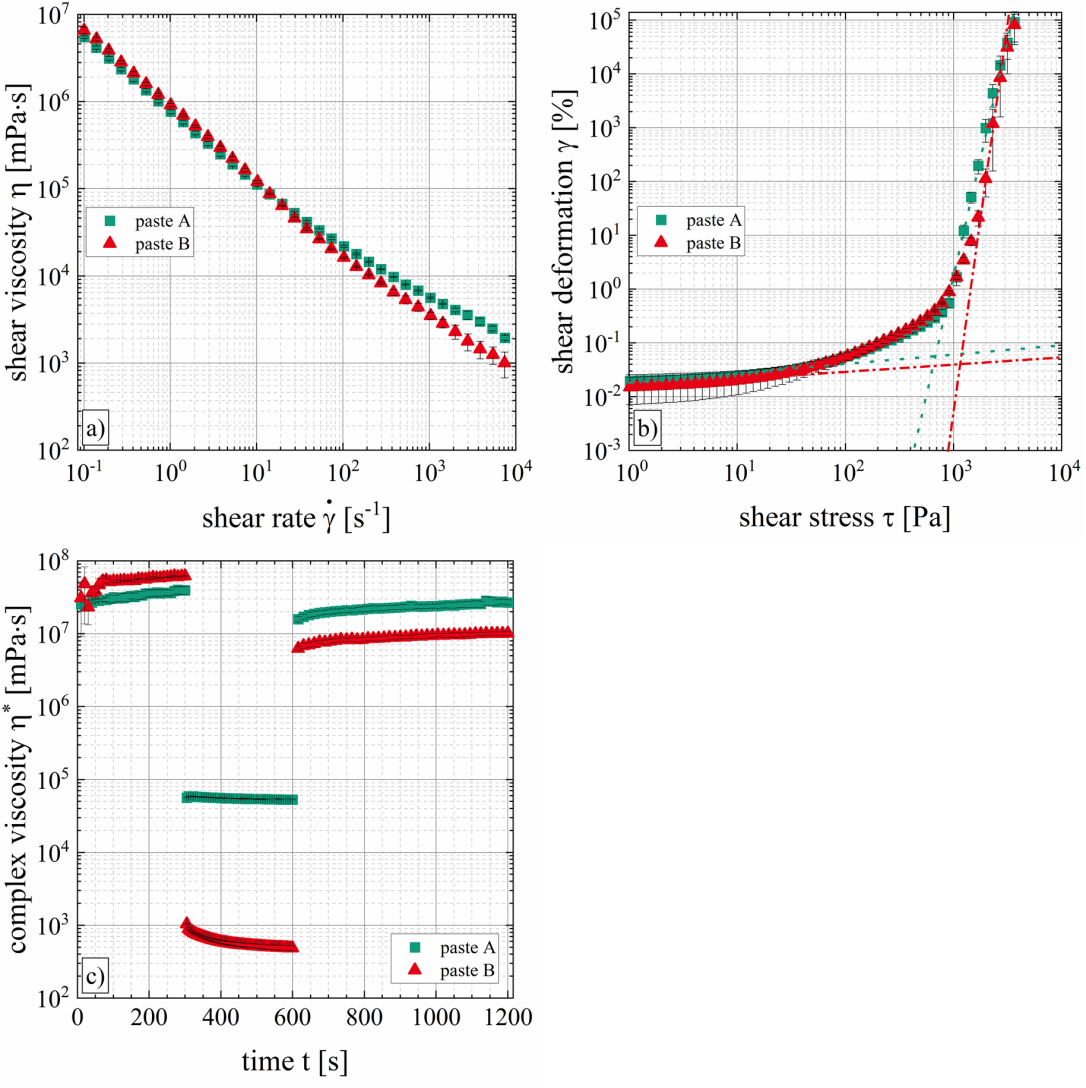
Shear rheological characterization of low-temperature curing Ag pastes A and B determined by TwinDrive rotational rheometer from Anton Paar GmbH, Germany, at T = 25 °C. (**a**) The shear viscosity η is plotted as a function of the shear rate $\dot{\gamma }$, showing shear-thinning behavior of both pastes. (**b**) The shear deformation γ over the shear stress τ is measured to determine the static yield stress τ_f_ using the tangent intersection method. (**c**) In addition, the result of the three interval thixotropy test is depicted.

Figure 3 shows the results of the rotational rheometer measurements. Both pastes show strong shear-thinning behavior meaning that the shear viscosity decreases with increasing shear rates. This rheological property is well established for metal pastes used in solar cell applications^38,39^. Paste A has a shear viscosity of η_paste-A_ = (111.9 ± 5.0) Pa s at a shear rate of $\dot{\gamma }$ = 10^1^ s^−1^ and a decreased shear viscosity of η_paste-A_ = (5.6 ± 0.1) Pa s at a shear rate of $\dot{\gamma }$ = 10^3^ s^−1^. Paste B shows a similar low shear viscosity (η_paste-B_ = (119.9 ± 4.6) Pa s at $\dot{\gamma }$ = 10^1^ s^−1^) (see Fig. 3a). For shear rates above $\dot{\gamma }$ > 100 s^−1^, the data suggests that both paste formulations show significant differences in shear viscosity from each other, indicating different behavior during printing because relevant process shear rates are between $\dot{\gamma }$ = 10^3^ s^−1^ and $\dot{\gamma }$ = 10^5^ s^−1^^6^. In literature, the phenomenon of edge fracture is well known for rotational rheometer measurements of highly filled suspensions^42,43^. This effect also occurred during our rotational measurements, hence the determined viscosity values for shear rates above $\dot{\gamma }$ > 100 s^−1^ might only give relative indications rather than absolute descriptions of the viscosity. Furthermore, wall slip, shear banding and sample spillage might affect the true shear viscosity values as described in ref.^32^.

The thixotropic recovery of the internal structure after excessive deformation is calculated by the last elastic storage modulus *G′* value of interval III and the last elastic storage modulus *G′* value of interval I as indicated in Fig. 3c). Here, the relative recovery of the storage modulus is calculated after a recovery time of t = 10 min to give an indication that describes the reconstruction of the inner paste structure. Paste A shows a relative recovery of r_paste-A_ = (69 ± 14)%, paste B of r_paste-B_ = (17 ± 1)%. Even though, a recovery time of t = 10 min does not correspond to the recovery time right after the printing process, it does give a general indication on the paste’s ability to recover its inner structure. The static yield stress of paste A is τ_f_ = (7.7·10^2^ ± 14) Pa, the static yield stress of paste B is τ_f_ = (1.2·10^3^ ± 140) Pa (see Fig. 3b). These rheological parameters under shear flow indicate that the low-temperature curing Ag pastes A and B have similar paste properties, but as we show in this study the applicable process velocities of category II (see “Definition of process velocity range resulting in homogeneous structures” section) and the corresponding Ag-electrode shapes (see “Impact of filament stretching on solar cell metallization” section) differ significantly. In the studies of Pospischil et al.^44^ and Tepner et al.^39^, the different Ag-electrode shapes were explicable by the shear rheological properties of high-temperature curing Ag pastes. These pastes were applied by parallel dispensing or flatbed screen printing.

#### Behavior under uniaxial extensional flow

**Figure 4:**
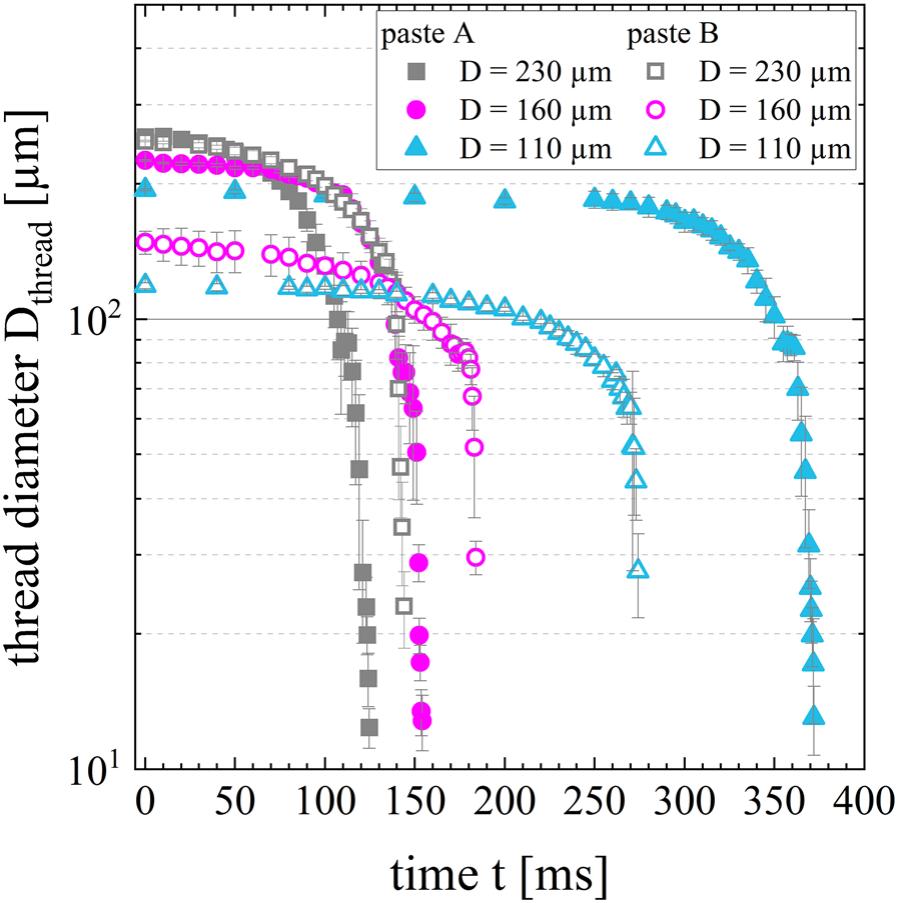
Necking of free-hanging extruded paste threads of pastes A and B. The mean thread diameter D_thread_ is plotted over time t for nozzle diameters of D = 230 µm, D = 160 µm and D = 110 µm. The curves show an exponential evolution.

Figure 4 illustrates the results of time-dependent filament necking of free-hanging paste threads for both low-temperature curing Ag pastes. At the beginning of extruding a paste thread, the filament diameter stays nearly constant. After the paste thread accumulated a critical mass for which the gravitational pull overcomes the yield stress of the suspension, the filament diameter changes abruptly. The necking of the paste threads evolves until it finally ruptures. Decreasing the nozzle diameter from D = 230 µm to D = 110 µm, increases the plateau of the curve because a longer extrusion time of the paste is required to achieve the critical mass of the paste thread to overcome the yield stress of the suspension. For paste A, the initial thread diameters are significantly larger than the nozzle diameter of D = 160 µm and D = 110 µm. In contrast, paste B does not show such a strong die swell; the initial thread diameter and the nozzle diameter are similar.

In Clasen’s study, the filament diameter as a function of the time is split into four different regimes. In this case, the elongational viscosity is determined by CaBER approach^45^. Based on this method, we defined two regimes for each curve in Fig. 4. The boundary condition is set as ten percent of the initial thread diameter. Therefore, the curve section including the exponential thinning is used to determine the apparent elongational viscosity *η*_*e,app*_*.* The graph shows an exponential trend generally described by the function D(t) = D_0_ + A·exp(R_0_·t). The derivative of the function *dD/dt* as well as the surface tension *Γ* are utilized to calculate the apparent elongational viscosity *η*_*e,app*_ (see Eq. (7)). The surface tension is assumed to be Γ = 20 mN m^−1^ for both formulations^6^.

**Figure 5:**
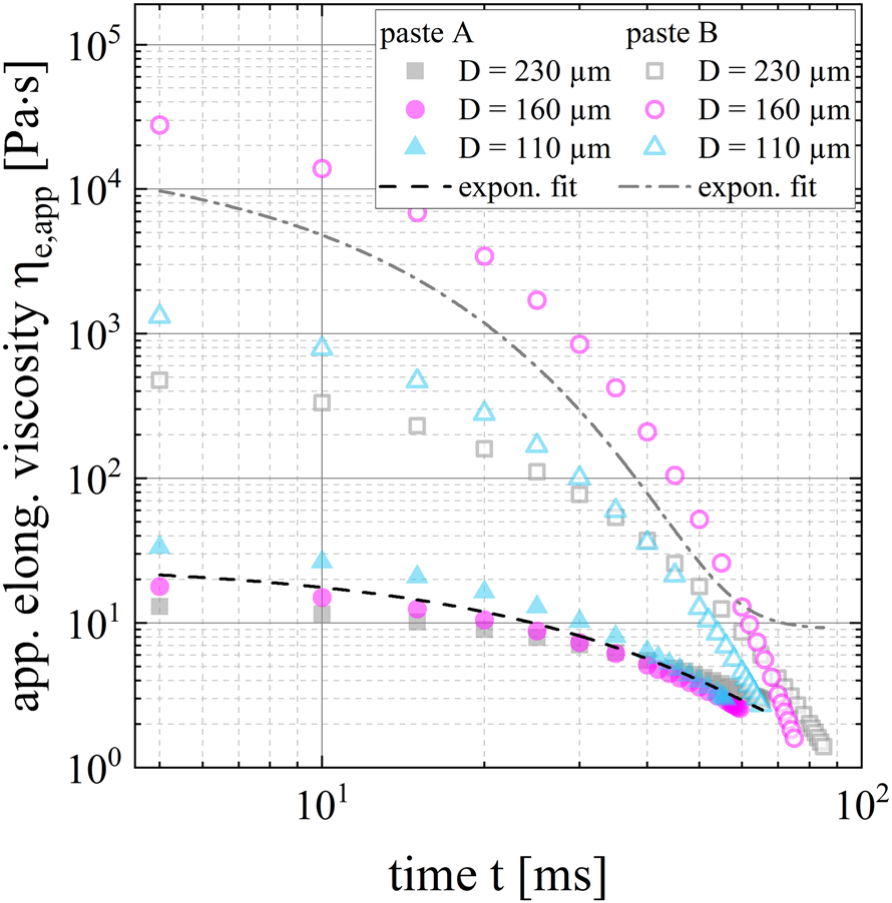
Apparent elongational viscosity of low-temperature curing Ag pastes A and B determined by filament necking at T = 25 °C. The elongational viscosity *η*_*e,app*_ is plotted as a function of time t.

The apparent elongational viscosities *η*_*e,app*_ over the time t and the resulting exponential fits for both pastes are depicted in Fig. 5. Paste A has an apparent elongational viscosity of η_e,app|paste-A_ = (18 ± 6) Pa s at t = 10 ms compared to an apparent elongational viscosity of η_e,app|paste-B_ = (4995 ± 5848) Pa s at t = 10 ms. This high apparent elongational viscosity of paste B is especially dictated by the measuring results of nozzle diameter D = 160 µm. The other data of paste B shows an apparent elongational viscosity of one order of magnitude less. No hypothesis explains these clearly different results between the nozzle diameters. Nevertheless, a clear difference in elongational viscosity of both low-temperature curing pastes and its exponential function can be detected. A steeper gradient of graph paste B corresponds to a stronger decrease in thread diameter. Following, the resistance to stretch the paste threads is less for paste A than paste B, resulting in a more excessive filament stretching for paste A. It is pointed out that the equations of the CaBER method are taken to determine the apparent elongational viscosity based on the raw data generated by the necking experiment.

### Definition of process velocity range resulting in homogeneous structures

**Figure 6:**
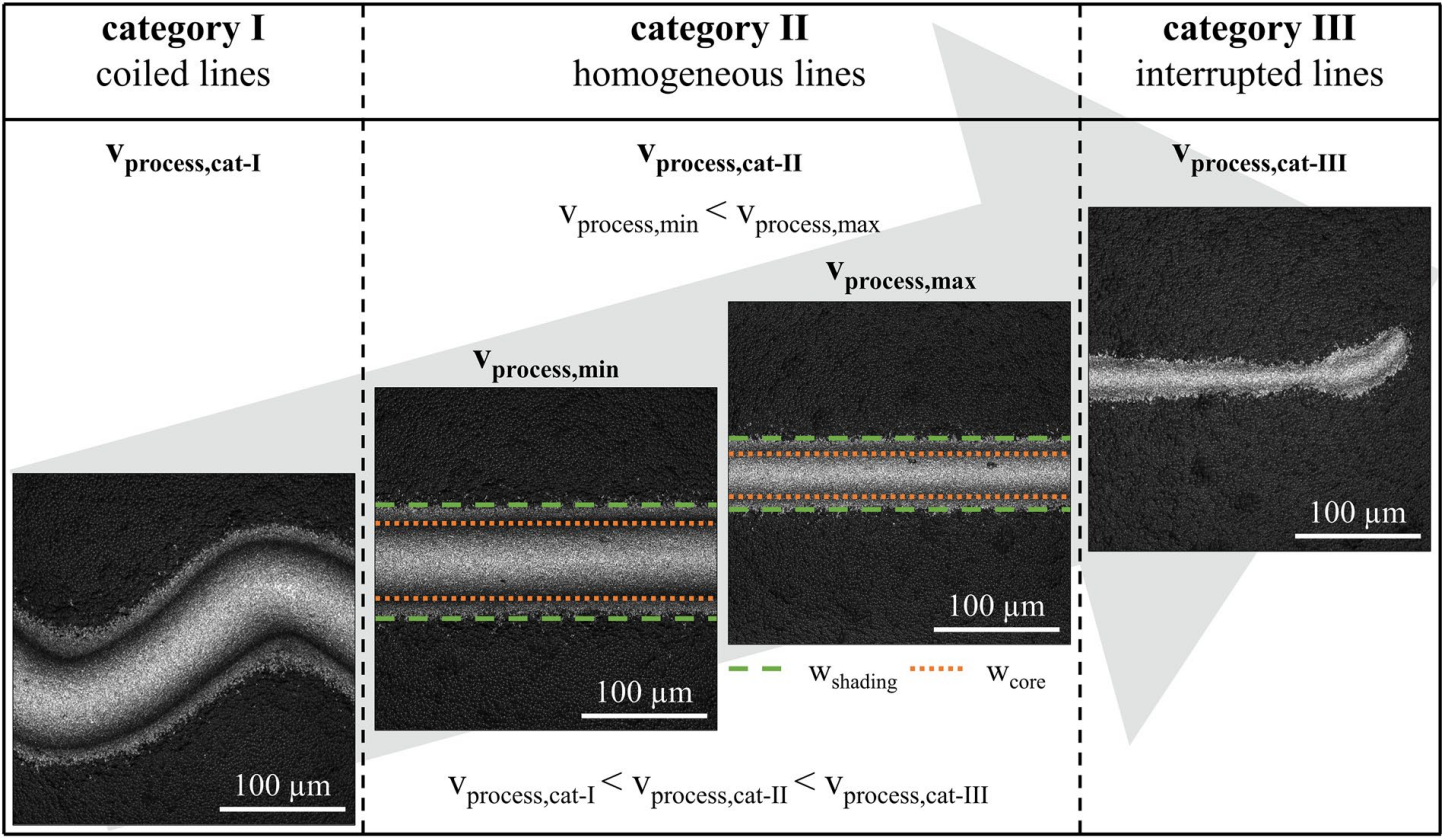
Classification of dispensed Ag-electrode shapes. Representative microscope images of different qualities of dispensed shapes of paste A (D = 45 µm) are depicted. Dispensing paste A with a process velocity of v_process,cat-I_ = 90 mm s^−1^ results in coiled lines (category I, left). For the process velocities between v_process,min_ = 140 mm s^−1^ (w_shading_ = 85 µm) and v_process,max_ = 410 mm s^−1^ (w_shading_ = 54 µm), homogeneous, straight line shapes with different widths are observed (category II, center). At a process speed greater than v_process,cat-III_ = 490 mm s^−1^ interrupted printing emerges (category III, right).

Extruding low-temperature curing Ag pastes A and B through micrometer nozzles result in different types of Ag-electrode shapes. Figure 6 illustrates the different dispensed line shapes, which we are using to categorize the dispensing result with the focus on solar cell metallization. These microscope images show examples of the printing results for paste A with a nozzle opening of D = 45 µm. When the applied process velocity is too slow for the specified process parameters, the printed line shape has a coiled pattern (category I) (see Fig. 6, left). This means that the volume flow rate of the paste is too high for the applied process velocity. In the center of Fig. 6, the process velocity is just right in order to achieve homogeneous, straight lines (category II). However, the homogeneous line electrode widths vary significantly depending on process velocity. Consequently, there exists a velocity range from minimal process velocity *v*_*process,min*_ to maximal process velocity *v*_*process,max*_ for which this type of low-temperature curing Ag pastes A and B show filament stretching during the printing. The minimal process velocity *v*_*process,min*_ is the lower limit for homogeneous straight line shapes, meaning that velocities below that critical threshold result in coiled lines whereas the maximal process velocity *v*_*process,max*_ defines the upper limit for printing of homogeneous, straight lines. Beyond that threshold, line interruptions emerge due to the excessive differences between the extrusion and the process velocity (category III) (see Fig. 6, right). Hence, the maximum process velocity *v*_*process,max*_ results in narrower line widths than the minimum process velocity *v*_*process,min*_. In the depicted example in Fig. 6, homogeneous straight line shapes could be dispensed in a process velocity range from v_process,min_ = 140 mm s^−1^ to v_process,max_ = 410 mm s^−1^. The shaded line electrode width *w*_*shading*_ varies between w_shading,min_ = 85 µm (w_core,min_ = 56 µm) and w_shading,max_ = 54 µm (w_core,max_ = 35 µm). It indicates that the extruded paste threads are lengthened significantly during the dispensing process.

**Figure 7:**
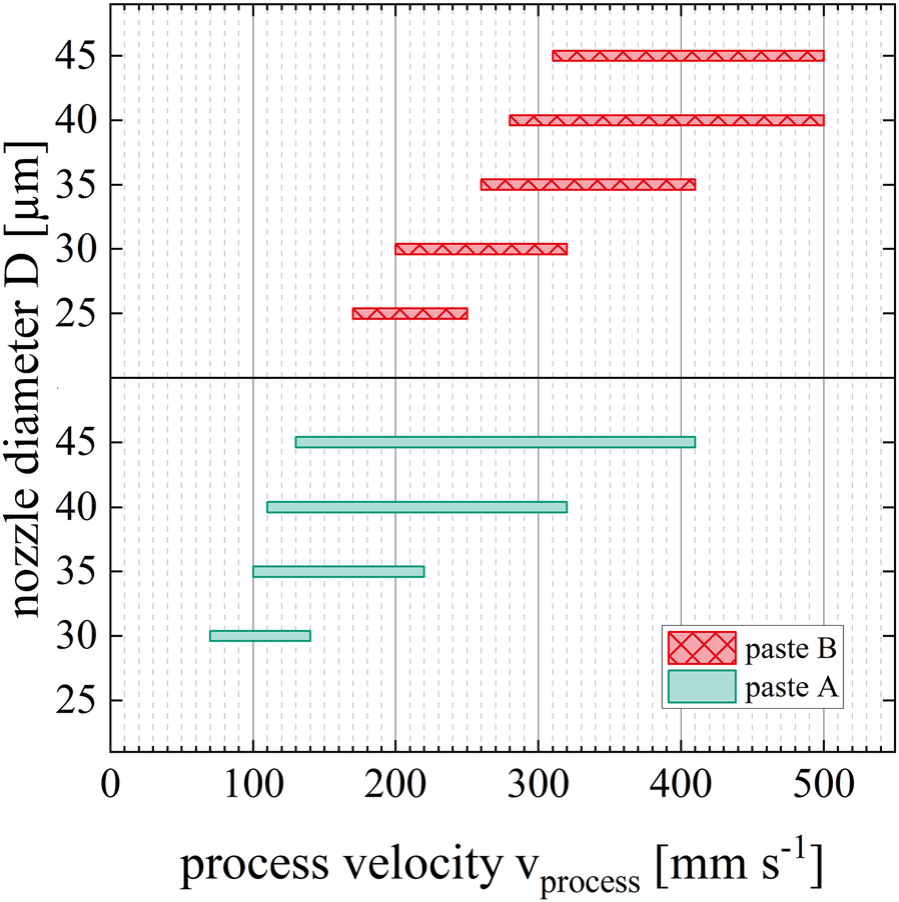
Process velocity ranges of category II for pastes A and B are presented for nozzle openings between D = 45 µm and D = 25 µm. The process velocity range is limited by the minimum process velocity *v*_*process,min*_ and the maximum process velocity *v*_*process,max*_.

Figure 7 depicts the process velocity range of category II relative to the nozzle diameter and paste formulation. Both low-temperature curing Ag pastes show the tendency that the applicable range of the process velocities shrinks with a reduction of the nozzle diameter. Paste A results in homogeneous, straight line shapes when it is extruded through nozzle openings of D = 45 µm with process velocities of v_process,min_ = 140 mm s^−1^ to v_process,max_ = 410 mm s^−1^. Process velocities below v_process_ < 130 mm s^−1^ at nozzle openings of D = 45 µm result in coiled lines. In contrast to that, process velocities above v_process_ > 410 mm s^−1^ result in interrupted line shapes. When paste A is extruded through nozzle openings of D = 30 µm, the process velocity range of category II decreases down to v_process,min_ = 70 mm s^−1^ and v_process,max_ = 140 mm s^−1^. Furthermore, paste A cannot be extruded through nozzle openings of D = 25 µm because of its particle size distribution and agglomerate sizes. On the other hand, paste B could be extruded up to process velocity values of v_process,max_ = 500 mm s^−1^ by using nozzle openings of D = 45 µm and D = 40 µm. Dispensing paste B through nozzle openings of D = 25 µm results in homogeneous, straight Ag-electrodes for process velocity values of v_process,min_ = 170 mm s^−1^ and v_process,max_ = 250 mm s^−1^. Therefore, paste B can be dispensed with faster process velocities compared to paste A, even when small nozzle diameters below D < 30 µm are used. This result is explainable by the different extrusion velocities v_extrusion_ of both highly filled suspensions (see “Impact of filament stretching on solar cell metallization” section, Fig. 9). One possible explanation of the different paste behaviors regarding the category II process velocity range might be the difference in polymer content and the different combinations of the two polymers in the paste’s formulations.

### Impact of filament stretching on solar cell metallization

**Figure 8:**
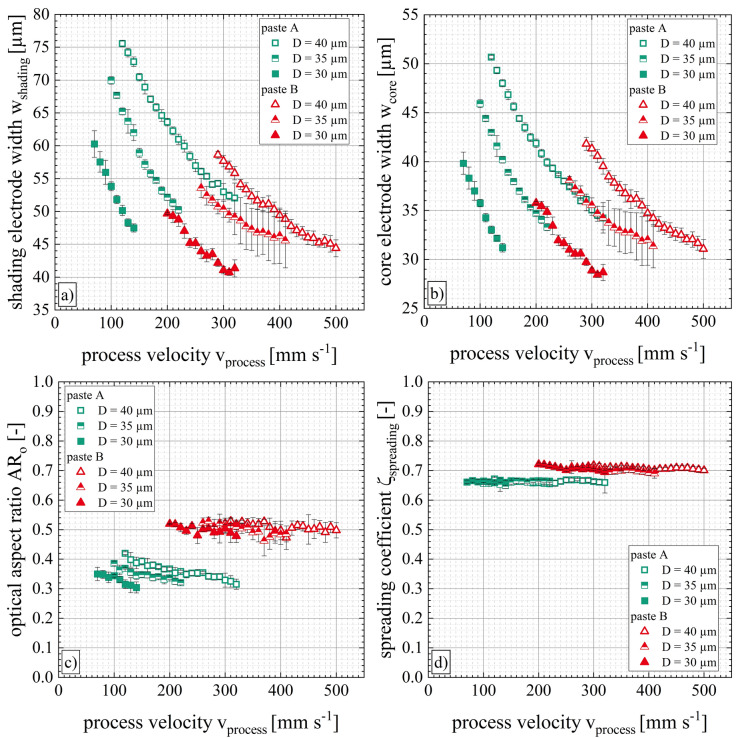
Ag-electrode shapes depending on homogeneous process velocities of category II for low-temperature curing Ag pastes A and B. The changes of (**a**) shading electrode width *w*_*shading*_, (**b**) core electrode width *w*_*core*_, (**c**) optical aspect ratio *AR*_*o*_, and (**d**) spreading coefficient *ζ*_*spreading*_ are depicted exemplarily for nozzle openings of D = 40 µm, D = 35 µm and D = 30 µm.

Figure 8 illustrates the impact of different process velocity ranges on the electrode geometry such as the shading electrode width *w*_*shading*_ (see Fig. 8a) and the core electrode width *w*_*core*_ (see Fig. 8b). The geometric variables which describe the line electrode shapes are given in ref.^6,39^. Here, the results of pastes A and B are shown for nozzle openings of D = 30 µm, D = 35 µm and D = 40 µm. Further results for additional nozzle diameters are summarized in Table 1. When extruding paste A through nozzle openings of D = 40 µm with the minimal process velocity of v_process,min_ = 120 mm s^−1^, the resulting shading electrode width is w_shading,paste-A_ = (76 ± 1) µm and the core electrode width is w_core,paste-A_ = (51 ± 1) µm. Increasing the process velocity up to v_process,max_ = 320 mm s^−1^, the shading electrode width as well as the core electrode width decrease approximately linearly. The same tendency can be observed with reduced nozzle diameters. However, the gradient of the relationship becomes steeper for smaller nozzle openings. The shading electrode width of paste A can be decreased from w_shading,paste-A_ = (60 ± 2) µm at v_process,min_ = 70 mm s^−1^ to w_shading,paste-A_ = (47 ± 1) µm at v_process,max_ = 140 mm s^−1^ for nozzle openings of D = 30 µm. One important observation is made, when using nozzle diameters of D ≥ 35 µm for paste A as the extruded paste threads can result in core electrode widths smaller than the corresponding nozzle openings. However, the corresponding shading electrode widths were always larger than the applied nozzle diameters regardless of the process velocity within category II. The significant die swell tendency of paste A also occurs in the dispensing process using small nozzle diameters which means that the initial paste thread diameter is larger than the nozzle diameters resulting in wider Ag-electrodes than the used nozzle diameters.

**Table 1:** Overview of process velocity ranges (category II) and resulting line shapes and Ag laydowns of low-temperature pastes A and B when dispensing the high-viscous suspensions through nozzle diameters of D = 25 µm to D = 45 µm.

	D [µm]	v_process,cat-II_ [mm s^−1^]		w_shading_ [µm]		w_core_ [µm]		AR_o_ [–]		ζ_spreading_ [–]		m_Ag_ [mg electrode^−1^]	
		v_process,min_	v_process,max_	min	max	min	max	min	max	min	max	min	max
paste A	45	140	410	85	54	56	35	0.43	0.33	0.66	0.66	1.90	0.71
	40	120	320	76	54	51	36	0.42	0.32	0.67	0.66	1.52	0.77
	35	100	220	70	50	46	33	0.39	0.32	0.66	0.66	1.19	0.58
	30	70	140	60	47	40	31	0.35	0.30	0.66	0.66	0.85	0.55
paste B	45	320	500	63	51	47	37	0.59	0.54	0.74	0.72	1.67	1.01
	40	290	500	59	44	42	31	0.51	0.50	0.71	0.70	1.26	0.73
	35	260	410	56	46	40	31	0.50	0.49	0.71	0.69	1.09	0.70
	30	200	320	50	41	36	29	0.52	0.48	0.72	0.69	0.82	0.58
	25	170	250	44	37	31	25	0.49	0.47	0.70	0.68	0.55	0.39

Paste B shows a similar tendency to paste A regarding the linear decrease of the shading electrode width and core electrode width when increasing the process velocity. Increasing the process velocity from v_process,min_ = 200 mm s^−1^ to v_process,max_ = 320 mm s^−1^ at D = 30 µm corresponds to an approximately linear decrease of the core electrode width from w_core,paste-B_ = (36 ± 1) µm to w_core,paste-B_ = (29 ± 1) µm. Nearly the entire process velocity range from *v*_*process,min*_ to *v*_*process,max*_ of paste B enables core electrode widths below the corresponding nozzle diameter.

Furthermore, Fig. 8 shows the evolution of the optical aspect ratio *AR*_*o*_ (see Fig. 8c) and the spreading coefficient ζ_spreading_ (see Fig. 8d) relative to the process velocity range of category II. The optical aspect ratio *AR*_*o*_ is defined as the ratio of the maximum electrode height *h*_*f,max*_ to the shading electrode width *w*_*shading*_ (see Eq. (8))^6^. The optical aspect ratio also decreases by increasing the process velocity, e.g. at the minimum process velocity v_process,min_ = 120 mm s^−1^ an optical aspect ratio of AR_o,paste-A_ = (0.42 ± 0.03) is achieved while the maximum process velocity v_process,max_ = 320 mm s^−1^ results in an optical aspect ratio of AR_o,paste-A_ = (0.32 ± 0.02) (D = 40 µm). The data suggests that when smaller nozzle openings are used, the obtainable optical aspect ratio decreases. While the decrease of the optical aspect ratio with an increasing process velocity is significant for paste A, this trend cannot be observed for paste B as the values remain almost constant.

The ratio of the core electrode width *w*_*core*_ to the shading electrode width *w*_*shading*_ is defined as the spreading coefficient ζ_spreading_ with the optimal value being ζ_spreading_ = 1 (see Eq. (9))^39^. The spreading coefficient of paste A is equal for all evaluated nozzle diameters and process velocities in category II (see Fig. 8d). However, the spreading coefficients of paste B show a minimal tendency depending on the process velocity which could be in the range of experimental uncertainty. Smaller spreading coefficients are reached with either increasing the process velocity or decreasing the nozzle diameter. Therefore, the data suggests that smaller nozzle diameters and higher process velocities may encourage paste spreading. This leads to the following hypothesis: for slow process velocities, the shear rheological paste properties dominate and the filament stretching does not occur to a significant extend. The minimum process velocity of paste B v_process,min_ = 170 mm s^−1^ reaches a spreading coefficient ζ_spreading,paste-B_ = (0.70 ± 0.03) and the maximum process velocity v_process,max_ = 250 mm s^−1^ shows a spreading coefficient ζ_spreading,paste-B_ = (0.68 ± 0.04) (D = 25 µm).

With regards to solar cell application, the applied metal grids should have narrow Ag-electrodes with no paste spreading and minimal silver consumption per cell in combination with high throughput rates, following high process velocities. Our data suggests that these goals follow a severe trade-off. The process velocity range of category II enables the reduction of the line electrode width as increasing process velocities result in decreasing core electrode widths as well as shading electrode widths. Using a smaller nozzle diameter helps to reduce the Ag-electrode width at the expense of significantly limiting the applicable process velocity while causing potential problems in process stability due to clogging of those nozzles because of the paste’s tendency to form agglomerates. In addition, smaller nozzle openings can encourage the paste spreading depending on the paste system. Nevertheless, dispensing low-temperature curing Ag pastes through small nozzle diameters results in the narrowest line electrodes.

Further, we have evaluated the Ag laydown per 156.75 mm line in relationship to the process velocity in category II (Table 1). For example, extruding paste A through nozzle openings of D = 45 µm results in a Ag laydown of m_Ag,paste-A_ = (1.90 ± 0.10) mg electrode^−1^, when using the minimal process velocity of v_process,min_ = 140 mm s^−1^. Increasing the process velocity up to v_process,max_ = 410 mm s^−1^ results in a decrease of paste laydown down to m_Ag,paste-A_ = (0.71 ± 0.03) mg electrode^−1^, following a delta reduction of Ag laydown by Δm_Ag,paste-A_ = − 1.19 mg electrode^−1^. The Ag saving potential of paste B in combination with nozzle diameters of D = 45 µm is only Δm_Ag,paste-B_ = − 0.66 mg electrode^−1^. Decreasing the nozzle diameter reduces the possible magnitude of Ag reduction as paste A shows a Ag laydown between m_Ag,paste-A_ = (0.85 ± 0.04) mg electrode^−1^ at v_process,min_ = 70 mm s^−1^ and m_Ag,paste-A_ = (0.55 ± 0.03) mg electrode^−1^ at v_process,min_ = 140 mm s^−1^ for 30 µm nozzle openings. Thus, filament stretching enables the control of Ag laydown per solar cell.

In conclusion, the maximum usage of filament stretching during micro-extrusion allows narrow Ag-electrodes and low Ag laydowns combined with high throughput potential. Consequently, the full usage of filament stretching should result in a gain in solar cell efficiency, as narrower Ag-electrodes decrease the shading area and thus increase the short-circuit current density. Further, the silver usage is more effective for homogeneous electrode shapes than inhomogeneous ones. We have evaluated this hypothesis in a different study, and the results are available in ref.^46^.

The question arises why low-temperature curing Ag pastes display such a vast process velocity range within category II allowing for such different electrode shapes as well as Ag laydown. The significant amount of polymer in the paste formulation might be one reason. For elastic materials like polymer-based suspensions, the elongational viscosity is reported as an influential property in the literature. During micro-extrusion, the paste is subject to uniaxial extensional deformation. Kunpai et al. demonstrated the different stretching of Ag pastes caused by varying amounts of graphite nanofibres. The resulting electrode widths were smaller than the applied nozzle openings because of the elongational properties of the pastes^13^. This elongational property of low-temperature curing Ag paste opens up the possibility to influence the Ag-electrode width and further the Ag laydown per cell without reducing the nozzle opening which can increase the process stability.

**Figure 9:**
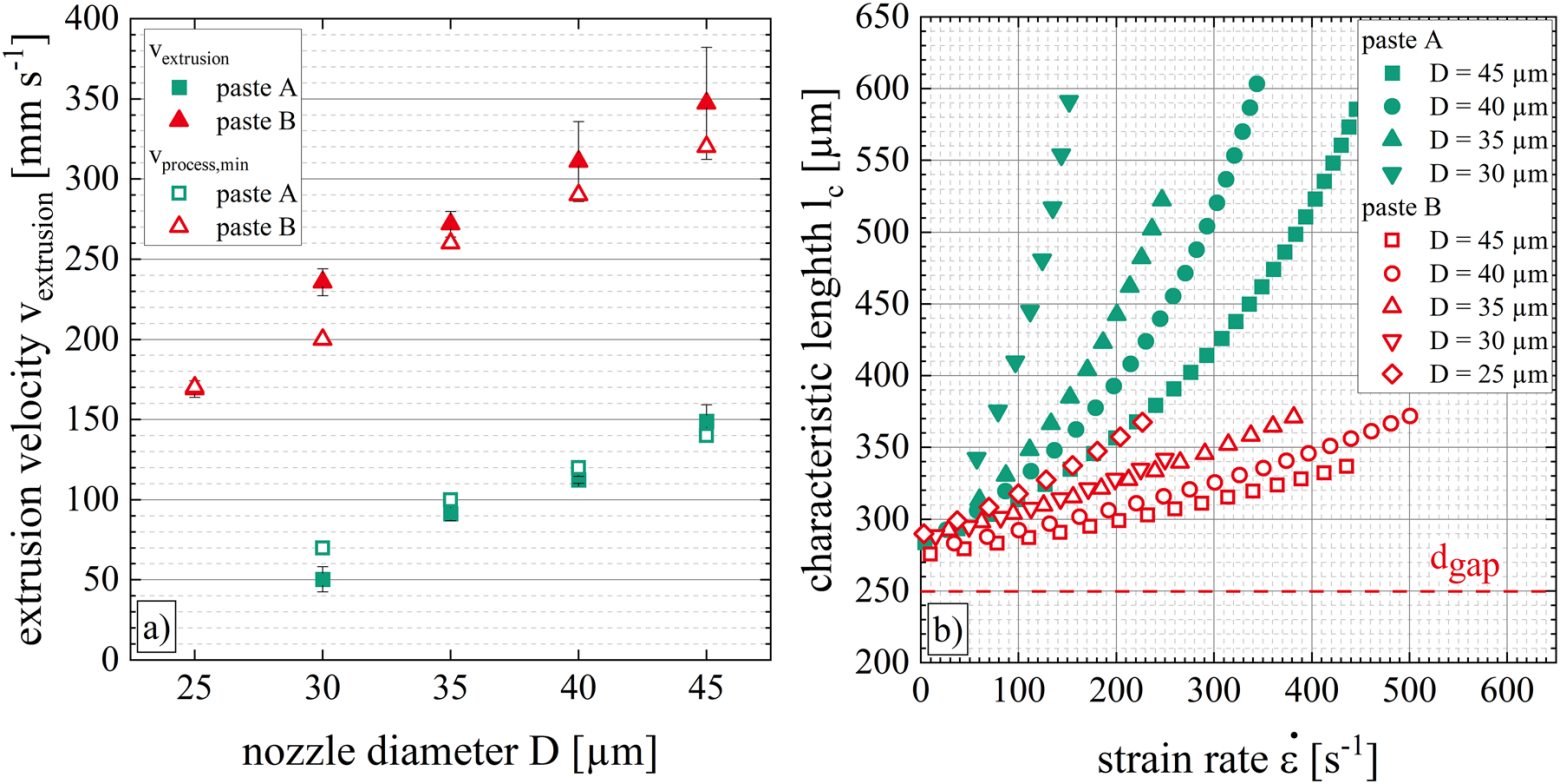
(**a**) Extrusion velocities *v*_*extrusion*_ for various nozzle diameters and (**b**) its characteristic length *l*_*c*_ over strain rate for low-temperature curing Ag pastes A and B. The extrusion of paste threads is observed by a high-speed imaging camera to determine the extrusion velocity *v*_*extrusion*_ for each paste depending on the nozzle diameter of 45 µm ≥ D ≥ 25 µm. Besides, the minimal process velocities v_process,min_ of Fig. 6 are shown to visualize our assumption v_process,min_ = v_extrusion_. The characteristic length *l*_*c*_ is calculated based on the equation in^13^.

Paste A has a lower elongational viscosity than paste B (see “Behavior under uniaxial extensional flow” section) which corresponds with the results of the printing experiment. The different electrode shape parameters as well as the different corresponding Ag laydown between the minimal and the maximal process velocity is higher for paste A than paste B, therefore the filament stretching of paste A appears to be greater. The lower elongational viscosity of paste A reflects a lower resistance regarding uniaxial extensional deformation which is also evident in the different characteristic lengths *l*_*c*_. Figure 9b) illustrates the characteristic length in dependence on the strain rate for different nozzle diameters. When the strain rate is $\dot{\varepsilon }$ = 0, the characteristic length *l*_*c*_ corresponds approximately to the corresponding dispensing gap *d*_*gap*_. This effect is independent of the nozzle diameter or paste formulation as the paste thread does not undergo any extensional deformation. Additionally, the measurement data allows the following assumption: v_process,min_ = v_extrusion_. The deviations between the two experimental data are within the range of experimental uncertainty and result partly from the chosen experimental parameters; for instance the minimal process velocity v_process,min_ is only determined in 10 mm s^−1^ increments. Increasing the strain rate results in an increased characteristic length, following the paste properties affect the characteristic length. Paste A shows a maximum characteristic length of l_c_ = 604 µm while paste B has a maximum characteristic length of l_c _= 372 µm. It seems that the characteristic length is independent of the nozzle diameter for the particular paste within experimental uncertainty. Even though paste B shows a significantly higher extrusion velocity compared to paste A, paste A has a higher stretching effect (see Fig. 9a)).

**Figure 10:**
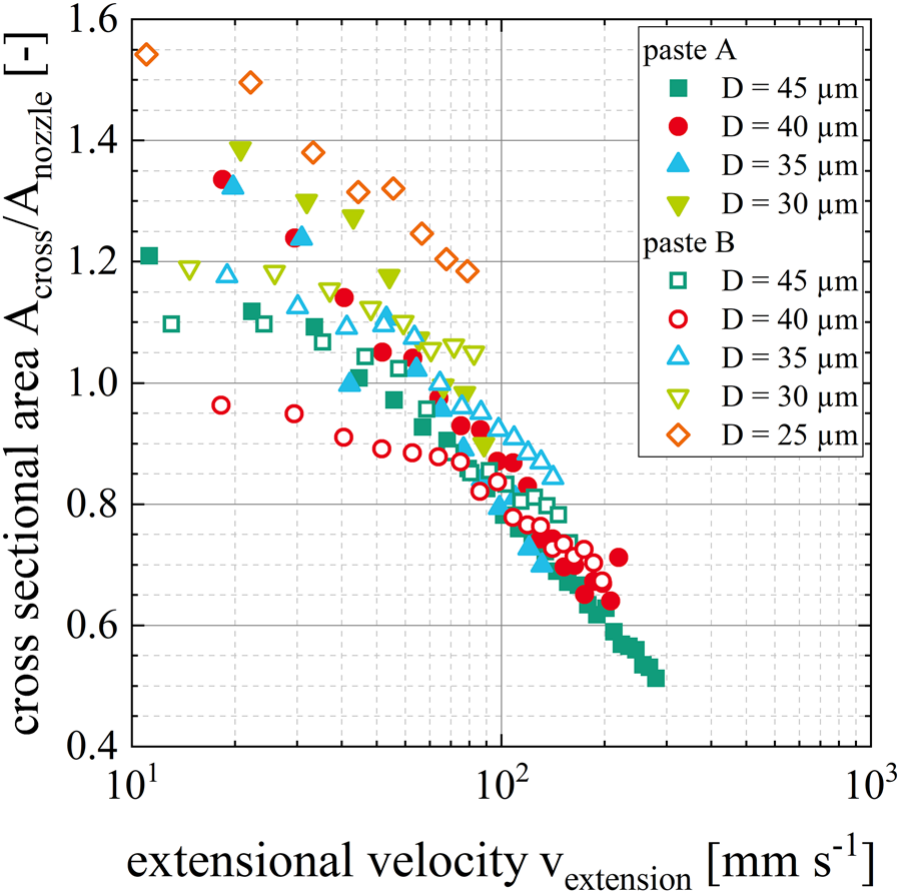
Correlation between elongation properties of the low-temperature curing Ag pastes A and B and the resulting electrode shapes. The ratio of cross-sectional area of the dispensed Ag-electrode *A*_*cross*_ to the cross-sectional area of the nozzle *A*_*nozzle*_ are plotted over the extensional velocity *v*_*extension*_.

Another link between the paste properties and the ratio of the cross-sectional area of the dispensed line *A*_*cross*_ to the cross-sectional area of the corresponding nozzle *A*_*nozzle*_ is presented in Fig. 10. The combination of different process velocities and extrusion velocities can result in the same extensional velocity, resulting in a comparable ratio of the two cross-sectional areas, especially for extensional velocities above v_extension_ = 10^2^ mm s^−1^ this relationship seems to appear to be independent of the suspension and the nozzle diameter. When the ratio of the cross-sectional areas becomes greater than one, the paste threads swell at the nozzle outlet or the wetting behavior of the paste onto the substrate becomes significant superimposing the filament stretching effect. When the ratio of the cross-sectional areas remains below one, the filament stretching effect dominates.

## Conclusion

In this study, we have demonstrated the filament stretching of low-temperature curing Ag pastes during micro-extrusion, enabling a significant decrease of line electrode width and Ag laydown by increasing the corresponding process velocities. In a series of rheological experiments, we have shown that two pastes with similar shear rheological properties differ significantly in elongational behavior. Further, the elongational viscosity for uniaxial extensional deformation for both pastes differs and reflects the degree of filament stretching and the potential characteristic length of the threads and its impact on the printing process.

The stretching effect allows to reduce the Ag-electrode width by down to Δw_f_ = − 40%_rel._ depending on the nozzle diameter and paste type. Further, a reduction of Ag laydown from m_Ag_ = 0.84 mg per Ag-electrode to m_Ag_ = 0.54 mg per line electrode for 30 µm nozzle openings has been demonstrated. These results show a promising way to further decrease the metallization costs while improving production throughput for SHJ solar cells and potentially enhancing solar cell performances. In order to achieve this, we suggest the development of new paste formulations which show a strong filament stretching effect and at the same time a low die swell tendency by adjustment of the polymer matrix.

## Data Availability

The datasets generated during and/or analysed during the current study are not publicly available due to an agreement between project partners in the project ALTURA with 03EE1006C number but are available from the corresponding author on reasonable request.
